# High-Precision Calibration Technology and Experimental Verification for Dual-Axis Laser Communication Systems

**DOI:** 10.3390/s25175233

**Published:** 2025-08-22

**Authors:** Wenyan Li, Xiaolei Zhang, Lei Zhang, Xiang Wei, Guoxi Luo, Peng Zhang, Zhipeng Xue

**Affiliations:** 1 Chang Guang Satellite Technology Co., Ltd., Changchun 130102, China; liwenyan@jl1.cn (W.L.); xuezhipeng@jl1.cn (Z.X.); 2 Department of Instrument Science and Technology, Xi’an Jiaotong University, Xi’an 100039, China

**Keywords:** satellite, laser communication, terminal, pointing accuracy, error correction

## Abstract

With the continuous improvement of remote sensing satellite resolution, laser communication technology has gained significant traction. The pointing accuracy of ground-based laser communication terminals is critical for the stability of satellite–ground laser transmission links. To enhance the pointing accuracy of ground-based laser communication terminals, this study proposes a high-precision calibration methodology utilizing an error correction mathematical model. This approach complements traditional methods. The pointing errors of an alt-azimuth dual-axis laser communication terminal system are analyzed, and the principles and implementation processes of the error correction mathematical model are presented. Calibration experiments were conducted using an existing laser communication terminal test platform. Observation error data were obtained by comparing stellar observations with theoretical stellar positions, and error model parameters were fitted. Verification through stellar observations after model establishment and error correction showed that the mean open-loop pointing error can be controlled to approximately 5″ or less. Compared to traditional methods, accuracy can be improved by over 85%, demonstrating significant and highly accurate error correction effects and validating the proposed method.

## 1. Introduction

In recent years, remote sensing satellites have entered a rapid development phase. As satellite resolution continues to improve, the volume of image data acquired daily by remote sensing constellations grows exponentially. The inability to transmit massive data promptly has become a bottleneck limiting the effectiveness of remote sensing satellites [1,2,3]. Compared to conventional microwave communication, laser communication offers advantages such as high bandwidth, low power consumption, and strong anti-interference capabilities, making it a core technology for future integrated space–ground communication and data transmission. Particularly for low-orbit small satellites with limited volume, weight, and power resources, equipping them with low-power, lightweight laser communication payloads is essential for increasing data transmission rates [4,5,6].

**Figure 1:**
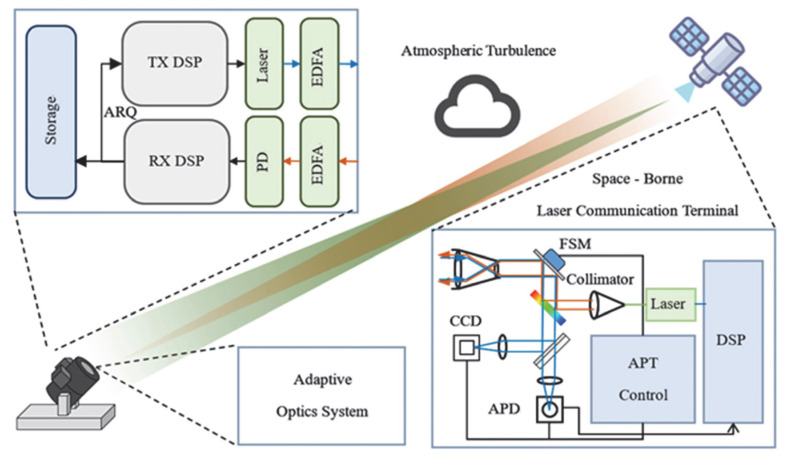
Schematic of satellite–ground laser communication system.

Due to the small divergence angle of laser beams and dynamic link instability, laser communication is susceptible to atmospheric disturbances, dynamic changes in weather conditions, and tracking errors, which may lead to frequent link interruptions and poor transmission stability. Currently, widespread application faces certain engineering challenges. Satellite–ground laser communication systems typically consist of onboard optical terminals, atmospheric channels, and laser terminals. Among these, the laser terminal plays a critical role, with technologies such as alignment, communication, and calibration being of utmost importance [7,8]. Composition of the a laser communication system is shown in Figure 1.

After establishing the satellite–ground link, the laser communication terminal must dynamically track the position changes in the in-orbit satellite through pointing adjustments to achieve communication. This imposes high requirements on the pointing accuracy of the terminal, making it a high-precision moving target tracking and measurement system. The axis types of such systems typically include alt-azimuth, equatorial, and horizontal configurations. Horizontal systems have no zenith blind zone and perform well in high-elevation tracking, but are rarely used due to mechanical constraints and limited installation space. Equatorial systems have their polar axis parallel to Earth’s rotation axis, allowing uniform rotation to compensate for stellar apparent motion caused by Earth’s rotation, making them suitable for astronomical observations. Alt-azimuth systems are widely used domestically and internationally due to their advantages such as large structural space, strong load capacity, compact structure, and low cost.

In 2014, NASA achieved information transmission to terminals via the Optical Payload for Lasercomm Science (OPALS) on the ISS, reaching 50 Mbps [9]. Real-time wavefront distortion correction via adaptive optics enhanced system Strehl ratio, laying groundwork for future LEO satellite high-speed optical communication [10]. In 2017, NASA’s Optical Communications and Sensor Demonstration (OCSD) project launched OCSD-B/C satellites, verifying feasibility of laser communication on microsatellites [11]. In 2018, IEEE comprehensively reviewed Acquisition, Tracking, and Pointing (ATP) mechanisms in Free-Space Optical (FSO) communication systems, listing pros/cons and discussing challenges/future directions [12].

In 2021, NASA launched the Laser Communications Relay Demonstration (LCRD) project, enabling bidirectional optical communication relay between GEO terminals and ground terminals/LEO satellites. LCRD integrated key technologies like adaptive optics, coding, link-layer protocols, and network-layer protocols [13,14,15]. In 2022, NASA and MIT’s terabyte Infrared Delivery (TBIRD) payload launched successfully. This <3 kg, 3U-sized terminal used commercial fiber transceivers with Wavelength Division Multiplexing (WDM) and dual-polarization Quadrature Phase Shift Keying (QPSK) to achieve 200 Gbps downlink. An uplink from the Optical Communication Telescope Laboratory (OCTL) provided Automatic Repeat Request (ARQ) feedback and tracking beacons, demonstrating significant potential for LEO-ground laser communication [16,17,18,19,20]. In 2024, NASA’s Deep Space Optical Communications (DSOC) project transmitted data from 226 million km, marking a key step toward interstellar networks [21]. In 2006, France’s airborne Laser Optical Link (LOLA) achieved laser communication between ARTEMIS satellite and Falcon 20 aircraft, validating feasibility for HAP-GEO links [7]. In 2010, Switzerland’s RUAG Space developed OPTEL-μ terminal enabling 2 × 1.25 Gbps communication [22,23]. In 2015, Germany’s DLR achieved 5.625 Gbps (LEO) and 1.8 Gbps (GEO) links via mobile terminals [24,25]. To meet growing LEO data demands, DLR initiated OSIRIS, using closed/open-loop pointing control; OSIRISv2 launched in 2016 at 1 Gbps [26]. In 2024, ESA plans to launch a satellite at Lagrange point L5 for 150 M km deep-space laser communication [27].

Laser terminals require rapid satellite acquisition and precise tracking (typically arc second-level). Despite progress, the literature rarely details pointing accuracy improvements. Beyond strict structural/assembly controls, older texts describe error decomposition/calibration. Traditional methods decompose errors into physical parameter models for correction, but effectiveness is limited. Errors are diverse/complex and cannot be fully eliminated. We also attempted error elimination using physical parameter models. Based on the test platform described in this paper, the error reached approximately 40″. High-precision system pointing calibration requires combining error analysis with software optimization.

This paper proposes a high-precision calibration technology based on an error correction mathematical model. First, system error analysis is conducted using an alt-azimuth laser communication terminal test platform to establish an error correction mathematical model. Second, the model is corrected through stellar observations to reduce system pointing errors. Finally, the effectiveness of the error correction and system pointing accuracy are verified through actual observation tests. The mean open-loop pointing error can be controlled to approximately 5″ or less. Compared to traditional methods, accuracy can be improved by over 85%.

## 2. Error Analysis

The laser terminal test platform employs an alt-azimuth dual-axis system, with pointing errors divided into azimuth axis error ΔA and elevation axis error ΔE:

(1) $$\begin{array}{l} \Delta A=\hat{A}-A=f(A,E)+\varepsilon_{A,i}\\ \Delta E=\hat{E}-E=g(A,E)+\varepsilon_{E,i} \end{array}$$

where *i* = 1, 2, …, *N* denotes the observation data index, *N* is the number of observations, $\hat{A}$ is the observed azimuth value, *A* is the theoretical azimuth value, $\hat{E}$ is the observed elevation value, *E* is the theoretical elevation value, $\varepsilon_{A,i}$ is the random observation error of the azimuth axis, and $\varepsilon_{E,i}$ is the random observation error of the elevation axis.

f(A,E) and g(A,E) are fitting correction models for azimuth and elevation pointing errors. Obtaining f(A,E) and g(A,E) allows pointing errors to be corrected via software methods [28,29,30,31,32,33,34,35].

Three-Axis Errors. The alt-azimuth structure has three main axes: azimuth axis, elevation axis, and collimation axis. The azimuth and elevation axes are mechanical rotation axes, while the collimation axis is the primary optical axis. The three axes should satisfy:

1. The azimuth axis is perpendicular to the horizontal plane.
2. The elevation axis is perpendicular to the azimuth axis.
3. The collimation axis is perpendicular to the elevation axis.

In practice, these axes deviate from ideal conditions, resulting in three-axis errors: collimation axis error, azimuth axis error, and elevation axis error.

Collimation Axis Error. The angle between the collimation axis and the perpendicular to the elevation axis is termed collimation axis error, denoted as *C*. Its impact on pointing accuracy is:

(2) $$\left\{\begin{array}{l} \Delta A_{C}=(\sec E_{M}-1)\cdot C\\ \Delta E_{C}=\frac{C^{2}}{2\xi }\tan E_{M} \end{array}\right.$$

where $\Delta A_{C}$ is the azimuth measurement error caused by *C*, $\Delta E_{C}$ is the elevation measurement error caused by *C*, *E_M_* is the elevation angle of the target, and ξ is the conversion coefficient from radians to arc seconds.

$\Delta E_{C}$ is a second-order small quantity in non-zenith measurements and can be neglected. Thus, collimation axis error primarily affects azimuth.

Azimuth Axis Error. The angle between the azimuth axis and the vertical line is termed azimuth axis error, denoted as *V*. The angle between the projection of the azimuth axis on the horizontal plane and the azimuth origin is termed azimuth axis tilt direction, denoted as $A_{H}$. Its impact is:

(3) $$\left\{\begin{array}{l} \Delta A_{I}=V\sin(A_{H}-A_{M})\tan E_{M}\\ \Delta E_{I}=V\cos(A_{H}-A_{M}) \end{array}\right.$$

where $\Delta A_{I}$ is the azimuth measurement error caused by *V*, $\Delta E_{I}$ is the elevation measurement error caused by *V*, and $A_{M}$ is the target azimuth angle.

### 2.1. Elevation Axis Error

The angle between the elevation axis and the perpendicular to the azimuth axis is termed elevation axis error, denoted as *b*. Its impact is:

(4) $$\left\{\begin{array}{l} \Delta A_{b}=b\tan E_{M}\\ \Delta E_{b}=\frac{b^{2}\tan E_{M}}{2\xi } \end{array}\right.$$

where $\Delta A_{b}$ is the azimuth measurement error caused by *b*, $\Delta E_{b}$ is the elevation measurement error caused by *b*.

### 2.2. Encoder Errors

The test platform uses encoders for angle measurement. Errors in azimuth and elevation encoders inevitably affect measurement accuracy [36,37,38,39].

Orientation Error. When the optical axis points north, the angle between the azimuth encoder zero line and the north direction is termed orientation error, denoted as *g*.

Zero-Position Error. When the optical axis is horizontal, the angle between the elevation encoder zero line and the horizontal plane is termed zero-position error, denoted as *h*.

Angle Measurement Error. This is the encoder’s deviation from the true value during operation and can be eliminated via error models.

### 2.3. Atmospheric Refraction

Due to atmospheric refraction, the observed direction of a celestial body differs from its true direction, known as atmospheric refraction. The true elevation is obtained by subtracting refraction from the observed elevation. Refraction increases at lower elevations and varies with temperature and pressure [40,41].

### 2.4. Other Errors

Minor effects are temporarily neglected.

## 3. Error Correction Mathematical Model Establishment

This paper proposes observing stars across the sky to obtain observed positions $\left(A_{M},E_{M}\right)$ and comparing them with theoretical positions $\left(A_{C},E_{C}\right)$ calculated from ephemerides to derive pointing deviations $\Delta A=A_{M}-A_{C}$, $\Delta E=E_{M}-E_{C}$. For given analytical expressions  f(A,E),  g(A,E) regression analysis and least-squares fitting are applied to fit coefficients in  ΔA=f(A,E),  ΔE=g(A,E).

The pointing error correction method involves conducting precise observations of target sources with known exact positions to obtain experimental samples of pointing errors. Mathematical methods are then used to solve for the correction coefficients of the pointing error correction model. These coefficients and the correction model are imported into the telescope control system, enabling the telescope to point more accurately at the target azimuth, thereby improving pointing accuracy.

The primary challenge is establishing the pointing error model, i.e., the analytical expressions for  f(A,E) and  g(A,E).

Two common models are physical parameter models and spherical harmonic models. Physical models have clear physical meanings but suffer from:

(1) Incomplete analysis of error sources, where errors caused by mechanical deformation, time delays, and environmental factors cannot be expressed by explicit functions;
(2) Small-angle approximation neglecting high-order terms;
(3) Assumption of independent errors (not strictly valid).

Spherical harmonic models ignore physical meanings, have low parameter correlation, and achieve higher accuracy and stability, Fitting parameters can effectively cover multiple errors. Thus, this paper adopts a fourth-order spherical harmonic model, i.e., a polynomial fitting equation characterizing all-sky pointing errors using spherical harmonics.

In the spherical harmonic model, zonal harmonics primarily describe axisymmetric slow-varying errors, such as systematic errors varying with elevation angle like tube gravity bending. Higher-order terms are needed for fitting. Tesseral harmonics describe azimuth-dependent periodic errors, such as axis clearance. First-order terms can cover the main components. Related studies show that the fourth-order spherical harmonic model offers better accuracy and stability than physical parameter models. The fourth-order zonal term can explain approximately 95% of the gravity deformation error. Increasing the order further improves accuracy by less than 2% [42]. Therefore, the zonal harmonics are taken to the fourth order, and the tesseral harmonics to the first order in the spherical harmonic model.

The principle of spherical harmonics involves expanding a function  f(θ,φ) on the spherical surface $S\;\left(i.e.,\;0\leq \theta \leq \pi ,0\leq \varphi \leq 2\pi \right)$ using Fourier series up to the fourth order:

(5) $$\begin{array}{c} f(\theta ,\varphi )=a_{0}+a_{1}\sin\varphi +a_{2}\cos\theta \cos\varphi +a_{3}\sin\theta \cos\varphi +a_{4}\sin^{2}\varphi +a_{5}\cos\theta \sin\varphi \cos\varphi \\ +a_{6}\sin\theta \sin\varphi \cos\varphi +a_{7}\cos2\theta \cos^{2}\varphi +a_{8}\sin2\theta \cos^{2}\varphi +a_{9}\sin^{3}\varphi \\ +a_{10}\cos\theta \sin^{2}\varphi \cos\varphi +a_{11}\sin\theta \sin^{2}\varphi \cos\varphi +a_{12}\cos2\theta \cos^{2}\varphi \sin\varphi \end{array}$$

Since a spatial model is established, the azimuth pointing error is a spatial pointing error. Using the secant principle, ΔAcosE replaces ΔA, i.e., ΔAcosE=f(A,E), ΔE=g(A,E). They are functions of azimuth angle *A* and elevation angle *E*. Substituting into the above equation yields the spherical harmonic error correction model:

(6) $$\begin{array}{c} \Delta A\cos E=a_{0}+a_{1}\sin E+a_{2}\cos A\cos E+a_{3}\sin A\cos E+a_{4}\sin^{2}E+a_{5}\cos A\sin E\cos E\\ +a_{6}\sin A\sin E\cos E+a_{7}\cos2A\cos^{2}E+a_{8}\sin2A\cos^{2}E+a_{9}\sin^{3}E\\ +a_{10}\cos A\sin^{2}E\cos E+a_{11}\sin A\sin^{2}E\cos E+a_{12}\cos2A\cos^{2}E\sin E \end{array}$$

(7) $$\begin{array}{c} \Delta E=b_{0}+b_{1}\sin E+b_{2}\cos A\cos E+b_{3}\sin A\cos E+b_{4}\sin^{2}E+b_{5}\cos A\sin E\cos E\\ +b_{6}\sin A\sin E\cos E+b_{7}\cos2A\cos^{2}E+b_{8}\sin2A\cos^{2}E+b_{9}\sin^{3}E\\ +b_{10}\cos A\sin^{2}E\cos E+b_{11}\sin A\sin^{2}E\cos E+b_{12}\cos2A\cos^{2}E\sin E \end{array}$$

## 4. Error Data Acquisition

### 4.1. Star Selection and Observation

Stars are used as references due to their fixed celestial positions. The sky is divided into zones: elevation from 30° to 75° in 15° intervals, azimuth in 20° intervals. At least 36 third-magnitude stars (one per zone) are observed sequentially.

### 4.2. Theoretical Star Position Calculation

Correct the mean right ascension and declination $\left(\alpha_{0},\delta_{0}\right)$ in the J2000 coordinate system for precession and proper motion parameters to obtain the mean position $\left(\alpha_{\mathrm{mean}},\delta_{\mathrm{mean}}\right)$ at the observation time:

(8) $$\begin{array}{l} \alpha_{\mathrm{mean}}=\alpha_{0}+(m+n\times \sin\alpha_{0}\tan\delta_{0})\times T+\mu_{\alpha }T\\ \delta_{\mathrm{mean}}=\delta_{0}+n\times \cos\alpha_{0}\times T+\mu_{\delta }T\\ m=3.0748575+0.0085330\times T-0.000000185\times T^{2}\\ n=20.043109-0.0085330\times T-0.00000217\times T^{2} \end{array}$$

where *μ_α_* is the century proper motion in right ascension, *μ_δ_* is the century proper motion in declination.

Apply nutation correction to $\alpha_{\mathrm{mean}}$, $\delta_{\mathrm{mean}}$ to calculate the true position $\left(\alpha_{\mathrm{true}},\delta_{\mathrm{true}}\right)$ of the star at the observation time (negative from mid-year to year-start, positive from mid-year to year-end):

(9) $$\alpha_{\mathrm{true}}=\alpha_{\mathrm{mean}}+(\Delta \psi +d\psi )\times (\cos\varepsilon +\sin\varepsilon \sin\alpha_{0}\tan\delta_{0})\times T-(\Delta \varepsilon +d\varepsilon )\cos\alpha_{0}\tan\delta_{0}$$

(10) $$\delta_{\mathrm{true}}=\delta_{\mathrm{mean}}+(\Delta \psi +d\psi )\times \sin\varepsilon \cos\alpha_{0}+(\Delta \varepsilon +d\varepsilon )\sin\alpha_{0}$$

where ε is the obliquity of the ecliptic:

(11) $$\varepsilon =23^{{}^{\circ}}26^{\prime }21.448^{\prime \prime }-46.915^{\prime \prime }\times T-0.00059^{\prime \prime }\times T^{2}+0.002813^{\prime \prime }\times T^{3}+(\Delta \varepsilon +d\varepsilon )$$

Apply annual aberration correction to the true position $\alpha_{\mathrm{true}},\delta_{\mathrm{true}}$ to obtain $\alpha^{\prime },\delta^{\prime }$ (caused by relativistic effects):

(12) $$\alpha^{\prime }=\alpha_{\mathrm{true}}-20.49552^{\prime \prime }\times (\cos L\cos\alpha_{0}\sec\delta_{0}+\sin L\sin\alpha_{0}\sec\delta_{0})$$

(13) $$\delta^{\prime }=\delta_{\mathrm{true}}-20.49552^{\prime \prime }\times [\cos L\cos\varepsilon (\tan\varepsilon \cos\delta_{0}-\sin\alpha_{0}\sin\delta_{0})+\sin L\cos\alpha_{0}\sin\delta_{0}]$$

(14) $$L=280{}^{\circ}27^{\prime }59.21^{\prime \prime }+129802771.36^{\prime \prime }\times T+1.093^{\prime \prime }\times T_{2}$$

Apply annual parallax correction to $\alpha^{\prime },\delta^{\prime }$ to obtain the final apparent position $\left(\alpha_{\mathrm{app}},\delta_{\mathrm{app}}\right)$ (caused by parallax from observing the same star from the Sun and Earth):

(15) $$\alpha_{\mathrm{app}}=\alpha^{\prime }-20.49552^{\prime \prime }\cdot (0.0532\pi \sec\delta_{0}\sin\alpha_{0}\cos L\cos\varepsilon -0.0448\pi \sec\delta_{0}\cos\alpha_{0}\sin L)$$

(16) $$\delta_{\mathrm{app}}=\delta^{\prime }-20.49552^{\prime \prime }\cdot [0.0532\pi \sec\delta_{0}\sin\alpha_{0}\cos L\cos\varepsilon -0.0448\pi (\tan\varepsilon \cos\delta_{0}-\sin\delta_{0}\sin\alpha_{0})\sin L]$$

### 4.3. Theoretical Observation Angle Calculation

To calculate pointing errors, the azimuth and elevation angles of the star in the measurement coordinate system must be determined.

First, calculate the true sidereal time. The expression for true sidereal time at Beijing observation time is:

(17) $$S=6^{h}41^{m}50.54841^{s}+8640184.812866\times T+0.093104^{s}\times T^{2}-6.2^{s}\times T^{3}+\left(\Delta \psi +d\psi \right)\times \cos\varepsilon$$

Given the station’s longitude *λ*, the hour angle t of the star relative to the station is:

(18) $$t=S+\lambda -\alpha_{\mathrm{app}}$$

The azimuth and elevation angles in the measurement coordinate system are:

(19) $$A=\tan^{-1}[\cos\delta_{\mathrm{app}}\sin t/(\cos\delta_{\mathrm{app}}\sin\psi \cos t-\sin\delta_{\mathrm{app}}\cos\psi )]$$

(20) $$E=\sin^{-1}(\sin\psi \cos\delta_{\mathrm{app}}+\cos\psi \cos\delta_{\mathrm{app}}\cos t)$$

where *A* is the theoretical azimuth angle, *E* is the theoretical elevation angle, and *λ*, ψ are the station’s longitude and latitude.

### 4.4. Atmospheric Refraction Correction

For general observations (non-low elevation angles), trigonometric functions can approximate atmospheric refraction. The empirical formula for atmospheric refraction (for any temperature and pressure) is:

(21) $$R=R_{0}(1+A+B)$$

where *R*_0_ is the atmospheric refraction at latitude 45° at sea level with temperature 0 °C and pressure 760 mm; *A* is the temperature variation coefficient at Celsius temperature *T*; *B* is the pressure variation coefficient at actual pressure *H* (in millimeters).

(22) $$A=\frac{-0.00383T}{1+0.00367T}$$

(23) $$B=\frac{H}{760}-1$$

The observed elevation angle is corrected to E−R. After correction, set the theoretical azimuth and elevation angles (with atmospheric refraction correction) as the CCD center, and observe the star using the equipment.

### 4.5. Centroid Calculation

After observing the star, the centroid method is used to calculate the captured spot to determine its precise center position. The grayscale of each point is threshold-corrected as:

(24) $$g_{2}(x,y)=\left\{\begin{array}{l} g(x,y)\;if\;g(x,y)>=g_{\tau }\\ 0\;if\;g(x,y)<g_{\tau } \end{array}\right.$$

The spot center is then expressed as:

(25) $$x_{0}=\frac{\sum_{i}\sum_{j}xg_{2}(x,y)}{\sum_{i}\sum_{j}g_{2}(x,y)},y_{0}=\frac{\sum_{i}\sum_{j}yg_{2}(x,y)}{\sum_{i}\sum_{j}g_{2}(x,y)}$$

Move the centroid to the CCD center, read $\hat{A}=A+\Delta A,\hat{E}=E+\Delta E$ from the sensor, and obtain ΔA and ΔE.

### 4.6. Excluding Outliers

Exclude anomalies where $\left|e_{i}\right|>3\sigma$, ensuring ≥24 valid error values.

## 5. Error Model Parameter Fitting

### 5.1. Test Platform Parameters and Meteorological Conditions

The telescope used in our test platform system is an alt-azimuth Schmidt-Cassegrain type. It is located at longitude 125.44°, latitude 43.79°, platform height 274 m. The aperture is 100 mm. The acquisition field of view is 260″ (elevation axis system). The acquisition CCD window is 1024 × 1024 pixels with a refresh rate of 20 Hz. The servo gimbal has a horizontal range ≥±270° and an elevation range of 0~90°. The angle measurement accuracy is better than 0.5”, the angle control accuracy is better than 5″, and the dynamic tracking accuracy is better than 1”. The gimbal collimation error is 0.02722°. We implemented temperature control for the CCD, ensuring its temperature remains within 20 ± 1 °C. A Normal Equations linear solver was employed.

Given the platform’s latitude of 43.79°, according to the atmospheric refraction theory presented, the influence of atmospheric pressure on pointing accuracy at this latitude is less than 0.1″. Conditions were not suitable for studying pressure’s impact. Therefore, pressure was not a primary factor in the experiments described; the average atmospheric pressure during the tests was used in calculations. Furthermore, the platform is situated in a dense forest. During testing, the forest canopy shaded the area, reducing solar radiation and turbulent exchange, resulting in very smooth humidity variations. Consequently, the impact of humidity on results could not be investigated, and humidity was not considered a primary influencing factor; the average humidity during the tests was used in calculations. Thus, this paper only discusses the influence of temperature changes on the results. Due to obstruction by surrounding trees, the test platform could only conduct experiments above 20° elevation.

The test platform system includes real-time communication-capable meteorological data logging equipment. It monitors temperature, pressure, humidity, etc., in real-time, usable for atmospheric refraction calculation, with an update frequency of once per hour. The refraction correction method used in this paper inputs the average meteorological parameters from each test into the refraction law. The average meteorological data for each test is shown in Table 1.

### 5.2. Star Selection and Test Procedure

For star selection, we referenced the FK5 catalog to choose 77 stars. The number of observed stars was increased based on practical conditions. The elevation range from 20° to 90° was divided into 7 equal parts. On the azimuth axis, to achieve uniform star distribution, it was divided into 16, 16, 15, 13, 10, 6, and 1 parts corresponding to the different elevation zones. Based on the telescope’s actual observation capability, stars of magnitude 5 or brighter closest to the center of each partition were selected.

However, suitable magnitude stars were not always available in planned airspace. Additionally, 3σ iterative truncation was used to remove measurement outliers (caused by inherent biases in the FK5 catalog). Consequently, fewer measurement points were retained during actual calibration than planned. When using 3σ truncation to remove outliers, σ was the standard deviation of the residuals before fitting. Values exceeding the 3σ range were considered outliers and removed. Iteration was performed multiple times until all data fell within the 3σ range of the final iteration.

**Figure 2:**
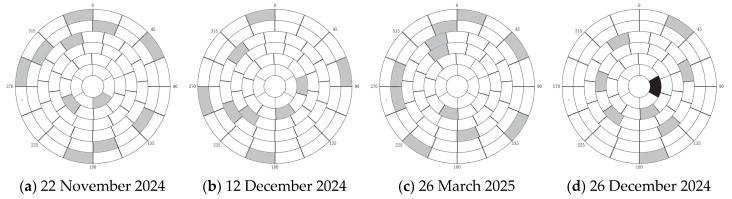
Star selection distribution map for fitting.

We conducted four tests on 26 March 2025, 22 November 2024, 12 December 2024, and 26 December 2024. Figure 2 shows the star selection status for these four tests. White represents regions with valid stars (one star per region), gray represents regions without valid stars, and black represents outlier regions.

After star selection, the theoretical positions of stars to be observed were first calculated. Theoretical observation azimuth and elevation angles were computed, and atmospheric refraction was added. Subsequently, stars were observed, centroids calculated, centroids moved to the CCD center, observed azimuth and elevation angles read, and pointing errors obtained. After obtaining multiple observation error values, 3σ truncation was used to remove measurement outliers. The remaining valid data was substituted into the spherical harmonic error model equations ΔA=f(A,E),ΔE=g(A,E) to derive the least-squares fitting polynomials for the azimuth and elevation error models, respectively. This served as the final error correction model. This model could then correct target observation positions during subsequent observation tasks.

## 6. Observation Experiment Results and Verification

### 6.1. System Error Correction

**Figure 3:**
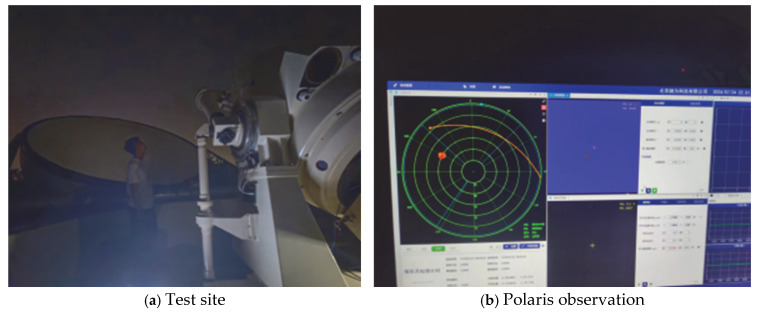
System error correction site diagram.

Calibration experiments were conducted using an alt-azimuth laser communication terminal test platform. Polaris was captured and observed, as shown in Figure 3a. The test site and Polaris imaging in the camera are shown in Figure 3b. Polaris is located within the middle 1/4 area of the entire field of view. The systematic deviations from the center field of view were 55″ in orientation error and 51″ in zero-position error. The imaging is circular with good spot quality. Therefore, this error can be written into the host computer for systematic compensation, enabling Polaris to be imaged at the center of the capture camera.

### 6.2. Stellar Observation and Calibration

**Figure 4:**
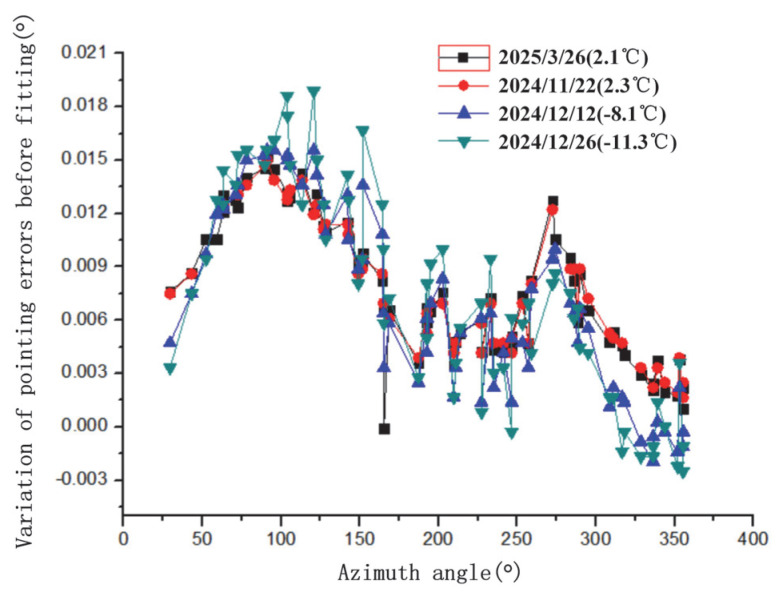
Variation in pointing errors with azimuth angle before fitting for four tests.

**Figure 5:**
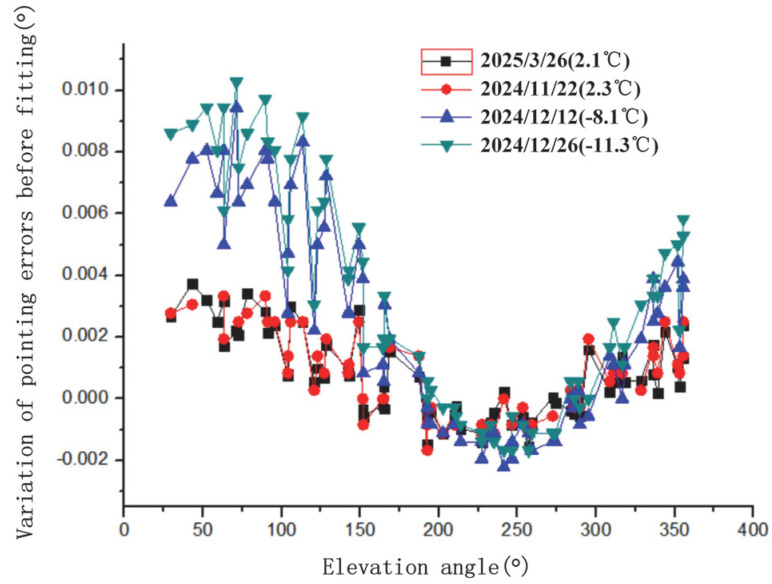
Variation in pointing errors with elevation angle before fitting for four tests.

Observe selected stars pre-fitting to obtain pointing errors. Pre-fit pointing errors vs. azimuth/elevation for four tests in Figure 4 and Figure 5. These errors include collimation, axis, encoder, and residual refraction errors.

Pre-fit error data substituted into spherical harmonic model to obtain coefficients. Coefficients applied to correct theoretical errors. Test data in Table 2.

From the test data, it is evident that each test converged after only one iteration using this method. Therefore, we conclude that σ (the standard deviation of residuals) was not reduced due to multiple iterations, indicating efficient model fitting without over-shrinking error bounds.

**Figure 6:**
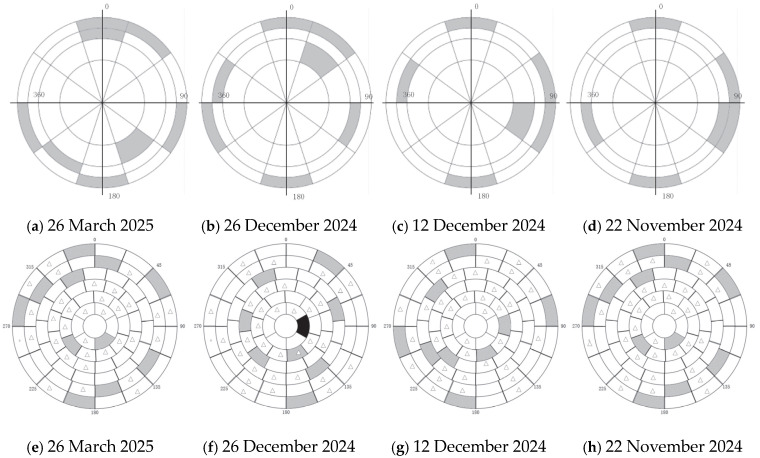
Star selection distribution map for verification.

Forty stars were randomly selected from the entire sky for observation verification tests. These stars were sourced from the FK5 catalog. Due to limitations in the test platform’s CCD camera capabilities, stars brighter than magnitude 5 were chosen to ensure measurement accuracy. The limited availability of qualifying stars resulted in significant overlap between the stars selected for verification and those used in fitting the spherical harmonic model, with an overlap rate of approximately 80%, The stellar regions used independently for verification are shown as gray areas in Figure 6a–d, while the overlapping portions with the stars selected for spherical harmonic fitting are shown as areas marked with triangle symbols in Figure 6e–h. As shown in Figure 6, the gray areas represent star regions excluded from spherical harmonic fitting, with each region containing one star.

**Figure 7:**
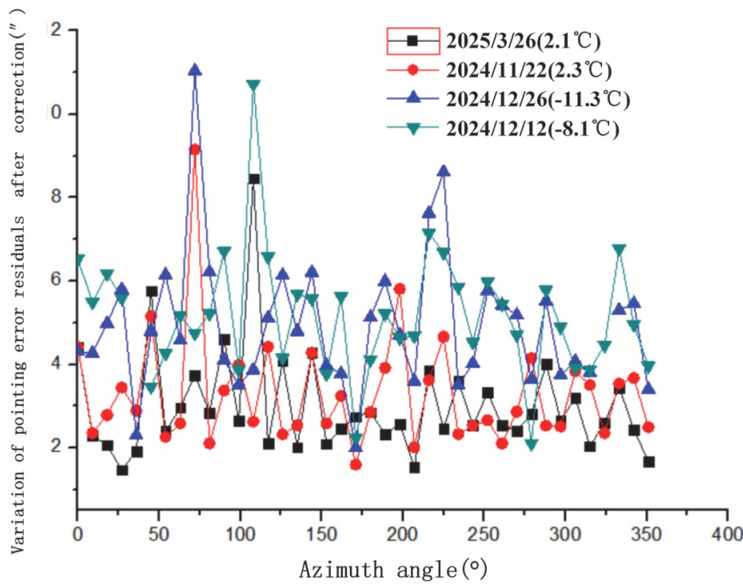
Variation in pointing error residuals with azimuth angle after spherical harmonic correction.

**Figure 8:**
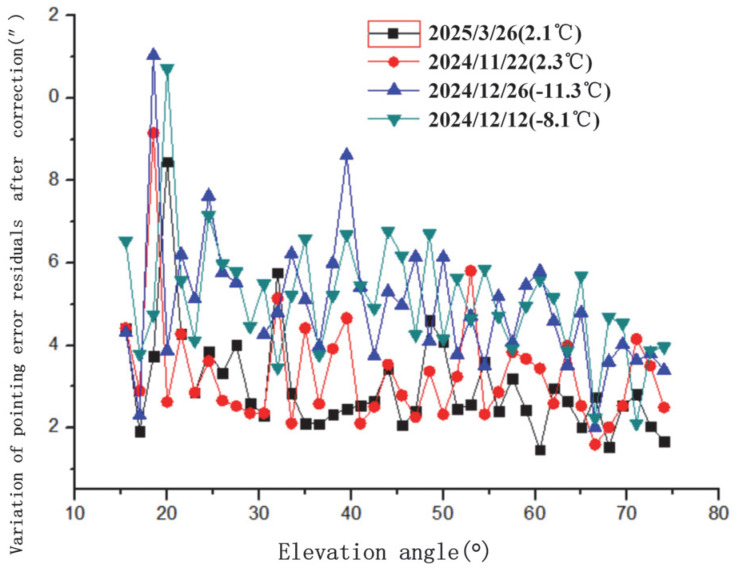
Variation in pointing error residuals with elevation angle after spherical harmonic correction.

**Figure 9:**
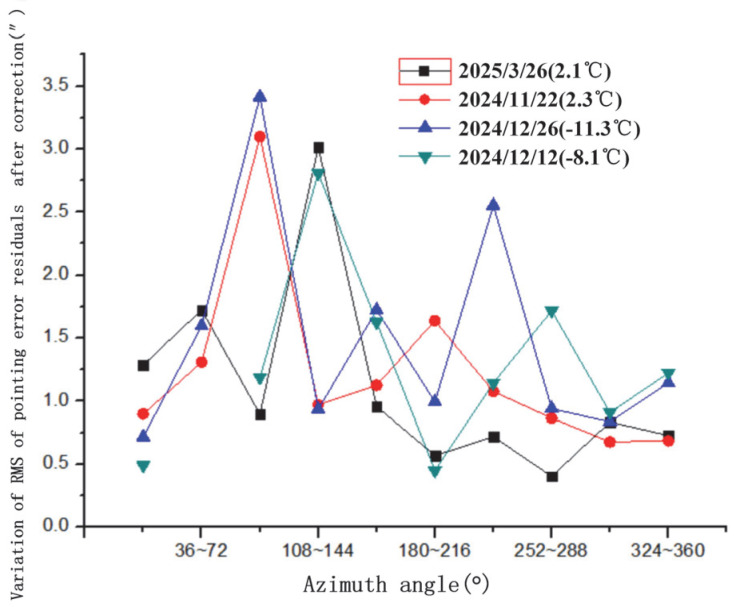
Variation in RMS of pointing error residuals with azimuth angle after correction.

**Figure 10:**
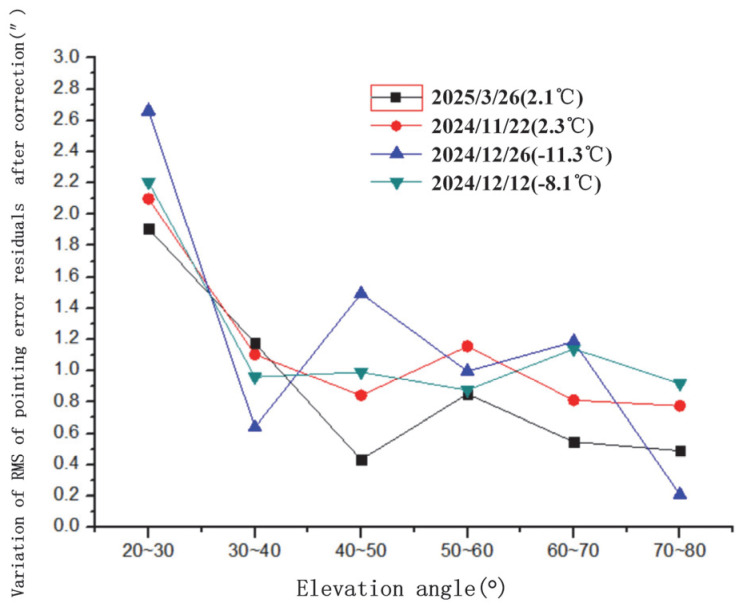
Variation in RMS of pointing error residuals with elevation angle after correction.

The variation in pointing error residuals with azimuth angle after spherical harmonic correction is illustrated in Figure 7. This figure shows the post-correction residual distribution, demonstrating the model’s effectiveness in reducing azimuth-dependent errors. The variation in pointing error residuals with elevation angle after correction is shown in Figure 8. This highlights how residuals change with elevation, with increased variability observed at lower elevation angles. The variation in the RMS (Root Mean Square) of pointing error residuals with azimuth angle after correction is displayed in Figure 9. The variation in RMS of pointing error residuals with elevation angle after correction is shown in Figure 10. This reveals an increase in RMS at lower elevations, particularly below 30°, indicating areas for potential refinement.

### 6.3. Results Analysis

Results indicate that temperature has a certain influence on pointing error, likely due to mechanical errors from thermal expansion/contraction. It is recommended to perform a new pointing calibration for every 3 °C temperature change. The stellar pointing calibration process for our test platform is fully automated, but each calibration takes approximately 2–3 h. Based on these results, stellar pointing error shows good reproducibility at the same temperature, and test repeatability is favorable. Therefore, assuming no significant long-term equipment drift, new tests can use calibration data from similar temperatures. Judging by the pointing error results, this approach is reasonable. The pointing error remains within acceptable limits when temperature changes are small, making it applicable in engineering operations.

The variation in pointing error residuals with azimuth and elevation angles is not pronounced. However, below 30° elevation, as elevation decreases to 20°, the residuals and their RMS tend to increase, with RMS rising more noticeably. Lower temperatures cause an increase in both the residual mean and RMS. Future work could involve larger sample sizes and long-term tests for observation and analysis.

**Figure 11:**
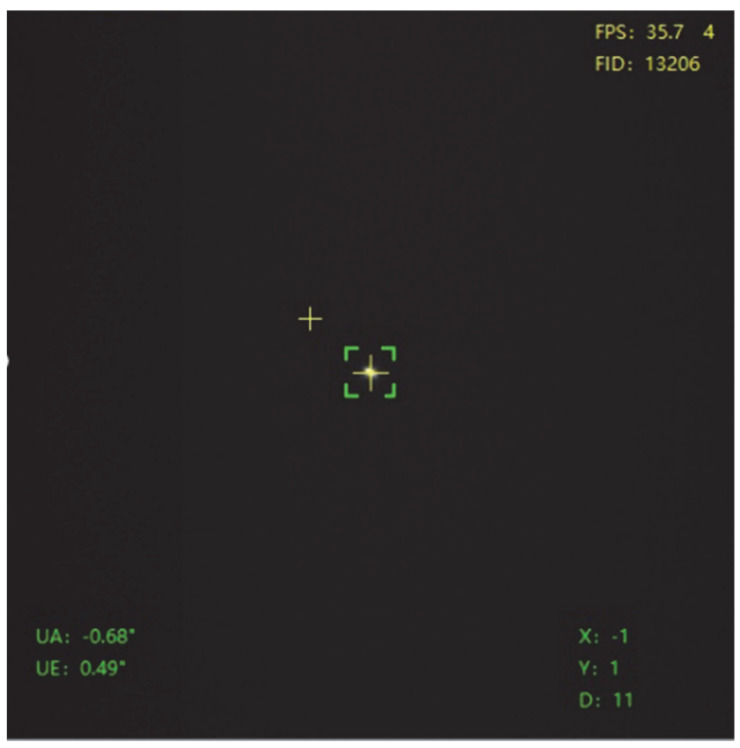
Revised Observational Target Visualization.

Overall, the mean pointing error post-correction was reduced to ≤5 arc seconds. The error correction effect is significant and highly accurate, validating the effectiveness of the proposed method. Observation results show that after model correction, the observation target is located at the center of the acquisition field of view under open-loop pointing conditions, as shown in Figure 11.

## 7. Conclusions

This paper proposes a method for calibrating the pointing accuracy of ground-based laser communication terminals by establishing an error correction mathematical model. First, mathematical expressions for various errors were derived through error analysis of an alt-azimuth dual-axis laser communication terminal system. Second, the principles for establishing the error correction mathematical model were elaborated. Theoretical stellar positions and observation angles were calculated, and stellar pointing deviations were obtained by comparing theoretical positions with actual observations. Regression analysis and least-squares fitting were applied to determine coefficients for the spherical harmonic correction model. Finally, verification experiments were conducted using a laser communication terminal test platform. After correcting orientation and zero-position errors, the laser communication terminal test platform was used for actual observations of selected stars to obtain observation errors. These were incorporated into the spherical harmonic error correction model. Subsequent stellar target observation verification showed the mean pointing error post-correction was reduced to ≤5 arc seconds. The open-loop accuracy significantly improved compared to pre-correction, validating the method’s effectiveness.

## Figures and Tables

**Table 1:** Average meteorological data per test.

	26 March 2025	22 November 2024	26 December 2024	12 December 2024
Temperature (°C)	2.1	2.3	−11.3	−8.1
Pressure (kPa)	97.8	98.3	91.6	90.6
Humidity (%)	76.3	76.2	72.1	73.1

**Table 2:** Test parameters and results for four stellar observations.

Item	26 December 2024	12 December 2024	22 November 2024	26 March 2025
Valid stars post-3σ truncation	68	68	63	66
Airspace without valid stars	8	9	14	11
number of anomalous stars 3σ-truncated	1	0	0	0
Iteration count	1	1	1	1
Post-correction mean residual (″)	4.93	5.16	3.3	3.0
Post-correction residual variance (″)	1.62	1.46	1.32	1.26
Post-correction residual RMS (″)	5.13	5.30	3.52	3.22
Temperature (°C)	−11.3	−8.1	2.3	2.1
Pressure (kPa)	91.6	90.6	98.3	97.8
Humidity (%)	72.1	73.1	76.2	76.3

## Data Availability

Data are contained within the article.
